# Mean Platelet Volume in a Series of 315 Patients with Rheumatoid Arthritis: Relationship with Disease Characteristics, including Subclinical Atherosclerosis and Cardiovascular Comorbidity

**DOI:** 10.3390/diagnostics13203208

**Published:** 2023-10-14

**Authors:** Marta González-Sierra, Alejandro Romo-Cordero, Juan Carlos Quevedo-Abeledo, Adrián Quevedo-Rodríguez, Fuensanta Gómez-Bernal, Antonia de Vera-González, Raquel López-Mejías, Candelaria Martín-González, Miguel Ángel González-Gay, Iván Ferraz-Amaro

**Affiliations:** 1 Division of Hospitalization-at-Home, Hospital Universitario de Canarias, 38320 Tenerife, Spain; martagses@gmail.com; 2 Division of Internal Medicine, Hospital Universitario de Canarias, 38320 Tenerife, Spain; alexromo96co@gmail.com (A.R.-C.); mmartgon@ull.edu.es (C.M.-G.); 3 Division of Rheumatology, Hospital Universitario Dr. Negrín, 35010 Las Palmas de Gran Canaria, Spainadrian-ce@hotmail.es (A.Q.-R.); 4 Division of Central Laboratory, Hospital Universitario de Canarias, 38320 Tenerife, Spain; fuensanta95@gmail.com (F.G.-B.); adeverag@gmail.com (A.d.V.-G.); 5 Epidemiology, Genetics and Atherosclerosis Research Group on Systemic Inflammatory Diseases, Hospital Universitario Marqués de Valdecilla, Instituto de Investigación Sanitaria Marqués de Valdecilla (IDIVAL), 39011 Santander, Spain; rlopezmejias78@gmail.com; 6 Internal Medicine Department, Universidad de La Laguna, 38200 Tenerife, Spain; 7 Department of Medicine and Psychiatry, Universidad de Cantabria, 39005 Santander, Spain; 8 Division of Rheumatology, IIS-Fundación Jiménez Díaz, 28040 Madrid, Spain; 9 Division of Rheumatology, Hospital Universitario de Canarias, 38320 Tenerife, Spain

**Keywords:** rheumatoid arthritis, mean platelet volume, cardiovascular disease

## Abstract

Mean platelet volume (MPV) refers to the average platelet size in femtoliters. Increased or decreased MPV has been associated with several disorders, including inflammatory and cardiovascular diseases. In the present study, our objective was to analyze the relationship of MPV with disease activity in a large and well-characterized series of patients with rheumatoid arthritis (RA). This is a cross-sectional study that included 315 patients with RA and 208 controls matched by sex and age. Complete blood count, including MPV, was assessed. Multivariable analysis was performed to examine the relationship of MPV with RA disease characteristics, carotid atherosclerosis, and traditional cardiovascular factors, including a comprehensive profile of lipid molecules and insulin resistance or beta cell function indices. The multivariable analysis, which includes other hematological modifications produced by the disease and platelet values, showed that MPV levels were significantly lower in RA patients than in controls. Erythrocyte sedimentation rate and interleukin-6, but not C-reactive protein, were negatively correlated with MPV after adjustment for covariates. Similarly, disease activity and MPV had a significant and independent negative correlation. No relationships were found between MPV and cardiovascular risk factors, lipid profile or insulin resistance indices or subclinical atherosclerosis. In conclusion, patients with RA have lower levels of MPV than controls. MPV is negatively related to acute phase reactants and disease activity in RA.

## 1. Introduction

The mean platelet volume (MPV) is the average platelet size. Like the mean corpuscular volume for red blood cells, it is determined as being the mean volume of circulating platelets in femtoliters (fL). The MPV normal value ranges between 6.0 and 13.2 fL [1], although the result may differ due to instrument-to-instrument variation or depending on the laboratory method used. Under normal circumstances, there is an inverse relationship between platelet size and number, as the total platelet mass rather than the platelet count is regulated by thrombopoietin [2]. Therefore, in the context of hematological conditions, an abnormal MPV may help to suspect the presence of a possible disease. In this sense, a high MPV may indicate the active production of platelets in the bone marrow, as occurs in immune thrombocytopenia or in some inherited disorders of platelet function. In contrast, a low MPV usually indicates bone marrow suppression, as in aplastic anemia [3].

MPV has been recognized to play a role beyond reflecting platelet activity [4]. In this regard, an increase in MPV was observed in patients with cardiovascular disease, stroke, respiratory diseases, chronic kidney failure, intestinal diseases, and diabetes. Specifically in cardiovascular disease, the MPV is associated with a higher risk of acute cardiac events [5], can be an independent high-risk factor of death after acute ischemic cardiac events [6], and it is a potent and independent predictor factor of an increased number of restenosis after cardiac angioplasty and higher mortality rate [7]. This also appears to be the case for cerebrovascular ischemia [8]. On the contrary, a decrease in MPV has been observed in patients with ulcerative colitis [9], tuberculosis [10], and carcinomas [11,12].

Rheumatoid arthritis (RA) is a symmetric, inflammatory, peripheral polyarthritis of unknown etiology. Common hematologic complications of RA include anemia, thrombocytosis, and cytopenias [13]. Other rare hematologic blood disorders in RA include Felty syndrome, large granular lymphocyte leukemia, or lymphoma. How MPV relates to disease characteristics in RA is controversial and most studies on this topic have been conducted in small groups of patients. For this reason, we have exhaustively characterized a large series of patients with RA. Information has been collected on disease activity parameters, immunological status, and comorbidity, including lipid profile, resistance to insulin action, and subclinical atherosclerosis. We have analyzed the relationship of all these characteristics with the MPV.

## 2. Materials and Methods

### 2.1. Study Participants

This was a cross-sectional study that included 315 consecutively recruited patients with RA and 208 sex- and age-matched controls. All patients with RA were 18 years old or older and fulfilled the 2010 ACR/EULAR classification criteria [14]. They had been diagnosed by rheumatologists and were periodically followed-up at rheumatology outpatient clinics. For inclusion in the present study, the duration of RA disease was required to be ≥1 year. Since glucocorticoids are often used in the treatment of RA, patients taking prednisone or an equivalent dose ≤ 10 mg/day were allowed to participate. Controls were community-based, recruited by general practitioners in primary care centers. However, controls with a history of any inflammatory rheumatic disease were excluded. None of the controls were receiving glucocorticoids. Patients and controls were excluded if they had a history of myocardial infarction, angina, stroke, a glomerular filtration rate < 60 mL/min/1.73 m^2^, a history of cancer or any other chronic disease such as hypothyroidism, heart or respiratory diseases, nephrotic syndrome, as well as evidence of active infection. None of the patients and controls had a hematological disease like aplasia or myeloproliferative disorders. The study protocol was approved by the Institutional Review Committee at Hospital Universitario de Canarias and at Hospital Universitario Doctor Negrín (both in Spain), and all subjects provided informed written consent (approval no. 2019-452-1). All research was performed in accordance with relevant guidelines/regulations and in accordance with the Declaration of Helsinki.

### 2.2. Data Collection and Laboratory Assessments

Individuals included in the study completed a questionnaire on CV risk factors and medication use and underwent a physical examination. Body mass index, BMI, (the weight in kilograms divided by the square of the height in meters), abdominal circumference, and systolic and diastolic blood pressure were assessed under standardized conditions. Information regarding smoking status, diabetes, and hypertension was obtained from the questionnaire. Medical records were reviewed to ascertain specific diagnoses and medications. The Sysmex-XN automated blood cell analyzer (Sysmex, Kobe, Japan) was used to measure blood cell counts. Cholesterol, triglycerides, and HDL-cholesterol were measured using an enzymatic colorimetric assay. LDL-cholesterol was calculated using the Friedewald formula. Dyslipidemia was defined if one of the following was present: total cholesterol > 200 mg/dL, triglycerides > 150 mg/dL, HDL-cholesterol < 40 in men or <50 mg/dL in women, or LDL-cholesterol > 130 mg/dL. A standard technique was used to measure the erythrocyte sedimentation rate (ESR) and high-sensitivity C-reactive protein (CRP). Human interleukin 6 (IL-6) was measured using an electrochemiluminescence immunoassay method (Roche Diagnostics, Indianapolis, IN, USA). The homeostatic model assessment (HOMA) method was performed to determine IR. Briefly, the HOMA model enabled an estimate of insulin sensitivity (%S) and β-cell function (%B) from fasting plasma insulin, C peptide, and glucose concentrations. In this study we used HOMA2, the updated-computer HOMA model [15]. Disease activity in patients with RA was measured using the Disease Activity Score (DAS28) in 28 joints [16], the Clinical Disease Activity Index (CDAI) [17], and the Simple Disease Activity Index (SDAI) [18]. DAS28-ESR and DAS28-CRP were categorized according to clinical remission (≤2.6), low disease activity (>2.6 and ≤3.2), moderate (>3.2 and 5.1), or high disease activity (>5.1) as previously described [19]. Similarly, SDAI categories were remission (<3.3), low disease activity (≥3.3 and ≤11), moderate disease activity (>11 and ≤26), and high disease activity (>26), and CDAI was categorized as remission (≤2.8), low (>2.8 and ≤10), moderate (>10 and ≤22), and high disease activity (>22) [20].

Cardiovascular risk score SCORE (Systematic COronary Risk Evaluation) 2 was calculated according to the 2021 European Society of Cardiology Guidelines on cardiovascular disease prevention in clinical practice [21]. SCORE2 risk categories are divided into low to moderate, high, and very high depending on different age groups (<50, 50–69 and ≥70 years). SCORE2 estimates an individual’s 10-year risk of fatal and non-fatal CV disease events in individuals aged 40 to 69 years. For healthy people aged ≥70 years, the SCORE2-OP (older persons) algorithm estimates 5-year and 10-year fatal and non-fatal CV disease events.

### 2.3. Carotid Ultrasound Assessment

Carotid ultrasound examination was used to assess carotid intima media thickness (cIMT) in the common carotid artery and to detect focal plaques in the extracranial carotid tree in only the patients with RA [22]. A commercially available scanner, the EsaoteMylab 70 (Genoa, Italy), equipped with a 7–12 MHz linear transducer and an automated software-guided radiofrequency technique, Quality Intima Media Thickness in real-time (QIMT, Esaote, Maastricht, Holland), was used for this purpose. As previously reported [22], based on the Mannheim consensus, plaque criteria in the accessible extracranial carotid tree (common carotid artery, bulb, and internal carotid artery) were defined as follows: a focal protrusion in the lumen measuring at least cIMT > 1.5 mm; a protrusion at least 50% greater than the surrounding cIMT; or arterial lumen encroaching >0.5 mm [23].

### 2.4. Statistical Analysis

Demographic and clinical characteristics in patients with RA were described as mean (standard deviation, SD) or percentages for categorical variables. For non-normally distributed continuous variables, data were expressed as median and interquartile range (IQR). Multivariable linear regression analysis, adjusting for confounders, was assessed to analyze the association between disease-related data and blood composite scores. Confounding variables were selected from demographics and traditional CV risk factors if they had a *p* value lower than 0.20 in the univariable relationship with MPV. All the analyses used a 5% two-sided significance level and were performed using Stata software, version 17/SE (StataCorp, College Station, TX, USA). *p*-values <0.05 were considered statistically significant.

## 3. Results

### 3.1. Demographic and Disease-Related Data

The demographic and disease-related characteristics of the participants are shown in Table 1. Most of the participants were women (80% in both populations, *p* = 0.45), with a mean age ± SD of 56 ± 17 years in controls and 54 ± 10 in the patients with RA (*p* = 0.17). The mean body mass index was slightly but significantly lower in RA patients than in controls. Classic cardiovascular risk factors were common in both patients and controls, but no significant differences were observed in the frequency of smoking, diabetes, hypertension, or dyslipidemia between both groups. Furthermore, the use of statins (*p* = 0.11) and aspirin (*p* = 0.081) did not differ between controls and patients (Table 1).

The median disease duration in this series of RA patients was 8 (IQR 4–14) years. The results of carotid ultrasound performed in patients with RA showed a mean cIMT of 0.677 ± 0.131 mm and 38% of them had carotid plaques. The mean CRP and ESR values in patients with RA at the time of the study were 2.9 (IQR 1.4–5.9) mg/L and 19 (IQR 9–38) mm/1st hour, respectively. Seventy percent of patients were positive for rheumatoid factor and 63% for anti-citrullinated protein antibodies. The disease activity measured by DAS28-ESR was 3.3 ± 1.3. Thirty-three percent of the patients were being treated with prednisone and eighty-three percent were taking at least one conventional disease-modifying antirheumatic drug of any type, with methotrexate being the most used (67%). Fifty percent of the patients were receiving antitumor necrosis factor therapies. The frequency of use of other treatments and historical data related to the disease are shown in Table 1.

### 3.2. Multivariable Analysis of Differences between Patients and Controls in Complete Blood Count

The values of cell blood count for controls and patients are shown in Table 2. Some differences were observed in the univariable analysis. In this regard, RA patients showed significantly higher levels of mean corpuscular red blood cell volume and mean corpuscular hemoglobin concentration, but lower red blood cell and lymphocyte counts, and a lower mean platelet volume. However, the number of platelets did not differ between RA patients and controls.

A first multivariable analysis (Model #1) was performed, which included all those demographic variables that differed between controls and patients with a *p* value less than 0.20 (age, body mass index, hypertension, diabetes, and the use of statins or aspirin). After this first adjustment, the significant differences observed in the univariable analysis were maintained. Furthermore, in order to rule out the confounding effect that other hemogram abnormalities could have on MPV, the difference between patients and controls in this parameter was additionally adjusted for platelets and for those cells counts or hematological parameters with a *p* less than 0.20 in the previous model (red blood cell count, mean corpuscular volume, mean corpuscular hemoglobin, and leukocytes and lymphocytes). After this adjustment (Model #2), MPV maintained a significantly lower value in RA patients compared to controls (beta coefficient −0.7 [95% confidence interval −1.1 to −0.3] fL, *p* = <0.001) (Table 2).

### 3.3. Relationship between Demographics and Disease Related to MPV in RA patients

The univariable and multivariable relationships of demographic and disease characteristics of RA patients with MPV are shown in Table 3. Age, sex, and all classic cardiovascular risk factors did not show significant associations with MPV. In contrast, ESR and IL-6, but not CRP, revealed significantly negative relationships with MPV after adjustment for covariates (those demographic variables or cardiovascular risk factors that had a *p* less than 0.20 in their univariable analysis). Moreover, patients taking methotrexate had significantly lower levels of MPV, while those on JAK-inhibitors exhibited a positive relationship with it after adjustment.

The disease activity indices are shown in Table 3 in a continuous manner and categorized as remission, low, moderate, and high disease activity. Furthermore, the moderate and high categories were merged into a single category due to the low number of patients with high activity in our series. When relationship scores were analyzed continuously, only DAS28-ESR showed a significant and independent relationship with lower levels of MPV. However, when this association was analyzed through categorical scores, the moderate and high categories of the DAS28-ESR and SDAI showed significantly lower levels of MPV compared to the reference category of remission. In the case of CDAI (*p* = 0.084) and DAS28-CRP (*p* = 0.071), statistical significance was not reached but a trend was observed (Table 3).

### 3.4. Relationship of Cardiovascular Risk Parameters to MPV in RA Patients

In general, no relationship was found between cardiovascular risk parameters and MPV (Table 4). In this sense, the cIMT, lipid profile, insulin resistance indices, and the presence of carotid plaques did not show a significant association with MPV. In the case of the SCORE2 calculator, patients with moderate cardiovascular risk showed significantly lower levels of MPV after multivariable adjustment. However, this was not the case for patients who were in the high-risk category (Table 4).

## 4. Discussion

Our study is the largest, to date, in which MPV has been studied in patients with RA. This has allowed us to perform a complete multivariable analysis considering possible confounding factors. According to our findings, patients with RA have lower levels of MPV than controls. In this regard, MPV decreases significantly and independently in patients with RA. A consistent negative association was also found between the activity of the disease and this hematological parameter.

MPV has been previously studied in RA but in smaller series and always using univariable analysis. These previous works have led to contradictory findings. In a study of 97 RA patients and 33 age- and sex-matched healthy subjects as a control group, MPV levels were significantly higher in RA patients in the univariable analysis. MPV was positively correlated with DAS28 score and decreased substantially after treatment with conventional and anti-TNF-alpha therapy [24]. On the contrary, in a study that included 261 patients with RA, a significant inverse correlation was found between MPV and ESR and CRP. Furthermore, in this series, the MPV was negatively correlated with the DAS-28-ESR/CRP [25]. Anti-TNF alpha therapy resulted in a significant increase in MPV at 2 weeks and 12 weeks in a report of 21 patients with RA [26]. In another study that included 60 consecutive RA, information on DAS28 score was assessed at baseline, and 2 months and 4 months after the admission time and beginning of the treatment schedule. Although disease activity decreased, no significant differences in MPV levels were observed at the three study time points. Furthermore, MPV measurement did not correlate with disease activity in RA patients within 4 months of treatment scheduling [27]. Finally, in a study that was carried out on 60 RA patients, MPV did not correlate with DAS28 score [28]. Despite this controversy, we believe that our study, due to the larger sample size, provides a better characterization of the patients, the ability to perform multivariable analysis, and allows us to draw more consistent and valid conclusions. 

In our work, a strong negative relationship was found between disease activity and MPV. This was found despite the use of different activity scores. It should be noted that the CDAI does not include acute phase reactants, while the SDAI is calculated using CRP and both versions of DAS28-ESR and DAS28-CRP are calculated with their respective acute phase reactants. Thus, regardless of the score used, and the acute phase reactant that the score includes, the relationship was eminently negative with all of them. In the case of DAS28-ESR, the relationship was strong. ESR is commonly assessed using the Westergren method, which measures the distance (in millimeters) at which red blood cells from anticoagulated whole blood fall to the bottom of an elongated, vertical, standardized tube over one hour due to the influence of gravity. For this reason, it is highly expected that ESR may be associated with hematological parameters such as MPV. On the contrary, CRP is measured in serum without being influenced, a priori, by hematological values. Despite this, DAS28-CRP also showed a negative relationship with MPV. This also applies to the SDAI, which includes the CRP in its calculation. Finally, the CDAI, which does not include any acute phase reactants, showed a tendency to be associated with MPV, although in this case it did not reach statistical significance. None of these facts were considered in previous works that denied the relationship between MPV and disease activity. In this sense, our study demonstrates a negative relationship between MPV and clinical disease activity, which turned out to be particularly strong when these relationships were also maintained after multivariable adjustment.

High values of MPV have also been linked to cardiovascular disease and cardiovascular events [5,6,7,8]. For this reason, we also studied its relationship with the presence of carotid atherosclerosis, and other cardiovascular risk determinants such as lipid profile and resistance to insulin action in RA. However, we could not find an association between MPV and cardiovascular disease in our series of patients with RA. The cross-sectional design of our study and the active treatment of our patients that led to remission or low disease activity in many of them may explain these negative results.

A mild to moderate increase in the platelet count, typically not exceeding two to three times the individual’s baseline, can be linked to the activity of RA [29]. Severe thrombocytosis may occur, often associated with extra-articular manifestations, particularly affecting the lungs, peripheral nerves, and blood vessels. In contrast, thrombocytopenia is rare in RA, except when it arises from medication or Felty syndrome. Surprisingly, in our study, the platelet count did not differ between patients and controls. As mentioned above, this may be because many patients had low or moderate disease activity. Nevertheless, although no difference was found, platelet count was included as a covariate in the multivariable analysis of the difference in MPV between groups. For this reason, it cannot be concluded, in any case, that MPV is lower in patients as a consequence of the number of platelets.

In our study, we found a significant negative relationship between IL-6 and MPV. IL-6 has been described to stimulate thrombopoiesis through thrombopoietin and has a role in inflammatory thrombocytosis [30,31]. The fact that MPV is inversely proportional to platelet count agrees with the relationship found in our work. Higher levels of IL-6 would likely lead to a higher platelet count, and, consequently, a lower MPV.

Our work may have several clinical implications. We believe that MPV would be useful for monitoring disease activity in RA. This is because MPV correlated with several types of disease activity scores and acute phase reactants. However, prospective studies are needed to define its usefulness in the response to treatments, or how its levels progress throughout the evolution of the disease. Despite this, MPV would not represent a reliable biomarker of cardiovascular disease in RA given its lack of relationship with traditional cardiovascular risk factors like dyslipidemia and insulin resistance, as well as SCORE2. Perhaps the particularities of cardiovascular disease in RA, which seem to be linked with systemic inflammation, are not mediated by this biomarker.

We acknowledge some limitations in our study. First, our study was cross-sectional and therefore causality cannot be inferred. Additionally, some of the patients were taking aspirin, which has antiplatelet action. However, the analysis of the difference in MPV between patients and controls was adjusted for aspirin. For this reason, we believe that our work controlled for the confounding effect that aspirin use could have had. Moreover, carotid ultrasound assessments were not available for controls. For this reason, we cannot conclude how MPV was related to carotid atherosclerosis in the control group.

In conclusion, MPV levels are decreased in patients with RA. In these patients, MPV is negatively related to acute phase reactants and disease activity.

## Figures and Tables

**Table 1:** Demographics, cardiovascular risk factors, and disease-related data in patients with RA and controls.

	Controls	Rheumatoid Arthritis	
	(*n* = 208)	(*n* = 315)	*p*
Age, years	56 ± 17	54 ± 10	0.17
Female, *n* (%)	162 (79)	254 (81)	0.45
BMI, kg/m^2^	**31 ± 3**	**28 ± 5**	**<0.001**
Cardiovascular risk factors and data			
Current smoker	35 (17)	56 (18)	0.78
Obesity	60 (29)	106 (34)	0.25
Hypertension	85 (41)	104 (33)	0.067
Diabetes Mellitus	39 (19)	43 (14)	0.10
Dyslipidemia	164 (79)	245 (78)	0.77
Statins, *n* (%)	58 (28)	109 (35)	0.11
Aspirin, *n* (%)	16 (8)	13 (4)	0.081
SCORE2, %	3.8 (1.2–9.3)	3.2 (1.6–5.6)	0.089
Carotid ultrasound			
cIMT, mm		0.677 ± 0.131	
Carotid plaque, *n* (%)		119 (38)	
Disease-related data			
Disease duration, years		8 (4–14)	
CRP at time of study, mg/L		2.9 (1.4–5.9)	
ESR at time of study, mm/1st hour		19 (9–38)	
IL-6, pg/mL		4.9 (3.2–8.2)	
Rheumatoid factor, *n* (%)		216 (70)	
ACPA, *n* (%)		193 (63)	
DAS28-ESR		3.34 ± 1.30	
DAS28-PCR		2.80 ± 1.05	
SDAI		13 (7–20)	
CDAI		8 (4–15)	
History of extraarticular manifestations, *n* (%)		30 (10)	
Bone erosions, *n* (%)		111 (39)	
Current drugs, *n* (%)			
Prednisone		104 (33)	
Prednisone doses, mg/day		5 (2.5–5)	
NSAIDs		139 (44)	
DMARDs		262 (83)	
Methotrexate		211 (67)	
Leflunomide		54 (17)	
Hydroxychloroquine		7 (2)	
Salazopyrin		2 (1)	
Anti TNF therapy		47 (15)	
Tocilizumab		19 (6)	
Rituximab		7 (2)	
Abatacept		6 (2)	
JAK inhibitors		16 (5)	

Data represent mean ± SD or median (IQR) when data were not normally distributed; CRP: C reactive protein. cIMT: carotid intima media thickness. NSAID: nonsteroidal anti-inflammatory drugs; DMARD: disease-modifying antirheumatic drug. TNF: tumor necrosis factor. ESR: erythrocyte sedimentation rate. SCORE: Systematic Coronary Risk Evaluation. JAK: Janus kinase. BMI: body mass index; DAS28: Disease Activity Score in 28 joints. ACPA: anti-citrullinated protein antibodies. Carotid ultrasound was not available for controls. CDAI: Clinical Disease Activity Index; SDAI: Simple Disease Activity Index. Dyslipidemia was defined if one of the following was present: total cholesterol > 200 mg/dL, triglycerides > 150 mg/dL, HDL cholesterol < 40 in men or < 50 mg/dL in women, or LDL cholesterol > 130 mg/dL. SCORE: Systematic COronary Risk Evaluation. Significant *p* values are depicted in bold.

**Table 2:** Multivariable analysis of differences between patients and controls in complete blood count cell count.

	Controls	RA Patients		Model #1		Model #2	
	(*n* = 208)	(*n* = 315)	*p*	Beta Coef. (95%CI), *p*			
	Univariable			Multivariable		Multivariable	
Red blood cells, ×10 10^6^/mm^3^	**4.71 ± 0.45**	**4.53 ± 0.39**	**<0.001**	**−0.2 (−0.3–(−0.1))**	**<0.001**		
Hemoglobin, g/dL	13.7 ± 1.4	13.6 ± 1.3	0.22				
Hematocrit, %	42.3 ± 3.8	43.8 ± 28.2	0.44				
Mean corpuscular volume of red blood cells, fL	**90 ± 6**	**92 ± 5**	**<0.001**	**1.9 (0.6–3.3)**	**0.005**		
Mean corpuscular hemoglobin, pg	**29 ± 2**	**30 ± 2**	**<0.001**	**0.8 (0.2–1.4)**	**0.005**		
Mean corpuscular hemoglobin concentration, g/dL	32 ± 1	34 ± 17	0.26	0.7 (−3–2−4.6)	0.72		
Leukocytes/mm^3^	7360 ± 1879	7114 ± 2109	0.17	−398 (−909–113)	0.13		
Neutrophils/mm^3^	4086 ± 1459	4065 ± 1595	0.87				
Lymphocytes/mm^3^	**2394 ± 829**	**2184 ± 818**	**0.004**	**−209 (−414–(−3))**	**0.046**		
Monocytes/mm^3^	583 ± 162	597 ± 207	0.44				
Eosinophils/mm^3^	232 ± 174	218 ± 164	0.35				
Basophils/mm^3^	49 ± 25	47 ± 44	0.48				
Platelets × 10^3^/mm^3^	264 ± 60	259 ± 63	0.40				
Mean platelet volume, fL	**10.3 ± 1.0**	**9.5 ± 1.6**	**<0.001**	**−0.7 (−1.1–(−0.3))**	**<0.001**	**−0.6 (−0.9–(−0.2))**	**0.001**

Data represent means ± SD or median (IQR) when data were not normally distributed. fL: Femtoliters. In the multivariable analysis, controls are considered the reference variable. Multivariable analysis Model #1 is adjusted for age, body mass index, diabetes, and statins and aspirin intake. Multivariable analysis Model #2 is adjusted for Model #1 plus red blood cells count, mean corpuscular volume, mean corpuscular hemoglobin, and leukocytes, lymphocytes, and platelets. Significant *p* values are depicted in bold.

**Table 3:** Demographic and MPV-related disease relationship in patients with RA.

	MPV, fL			
	Beta Coefficient (95%CI), *p*			
	Univariable		Multivariable	
Age, years	0.001 (−0.02–0.02)	0.89		
Female, *n* (%)	0.4 (−0.07–0.8)	0.097		
BMI, kg/m^2^	0.004 (−0.03–0.04)	0.83		
Cardiovascular risk factors				
Current smoker	0.3 (−0.2–0.8)	0.20		
Obesity	0.06 (−0.3–0.4)	0.74		
Hypertension	−0.3 (−0.7–0.1)	0.14		
Diabetes Mellitus	0.2 (−0.3–0.8)	0.35		
Dyslipidemia	−0.2 (−0.6–0.03)	0.45		
Statins, *n* (%)	0.04 (−0.3–0.4)	0.82		
Aspirin, *n* (%)	−0.07 (−0.8–0.6)	0.85		
Disease-related data				
Disease duration, years	−0.002 (−0.02–0.02)	0.84		
CRP, mg/L	0.0008 (−0.01–0.01)	0.90		
ESR, mm/1st hour	**−0.02 (−0.02–(0.007))**	**<0.001**	**−0.02 (−0.02–(−0.007))**	**<0.001**
IL-6, pg/mL	**−0.02 (−0.04-(−0.00009))**	**0.049**	**−0.02 (−0.04-(−0.0009))**	**0.040**
Rheumatoid factor, *n* (%)	0.2 (−0.2–0.6)	0.37		
ACPA, *n* (%)	**0.4 (0.02–0.8)**	**0.039**	**0.4 (−0.005–0.7))**	**0.047**
DAS28-ESR	**−0.2 (−0.3–(−0.06))**	**0.006**	**−0.2 (−0.3–(−0.06))**	**0.004**
Remission, *n* = 102	ref.		ref.	
Low, *n* = 55	−0.2 (−0.7–0.3)	0.49	−0.2 (−0.7–0.3)	0.51
Moderate, *n* = 118	**−0.6 (−1–(−0.2))**	**0.007**	**−0.6 (−1–(−0.1))**	**0.008**
High, *n* = 35	−0.4 (−10.2)	0.22	−0.5 (−1–0.2)	0.15
Moderate and high, *n* = 153	**−0.5 (−0.9–(−0.1))**	**0.009**	**−0.5 (−0.9–(−0.1))**	**0.008**
DAS28-PCR	−0.1 (−0.3–0.07)	0.24		
Remission, *n* = 154	ref.		ref.	
Low, *n* = 55	−0.06 (−0.6–0.4)	0.80	−0.03 (−0.5–0.5)	0.92
Moderate, *n* = 88	**−0.5 (−0.9–(−0.06))**	**0.027**	**−0.5 (−0.9–(−0.06))**	**0.026**
High, *n* = 11	−0.5 (−0.5–1)	0.34	0.5 (−0.5–1)	0.36
Moderate and high, *n* = 99	−0.08 (−0.6–0.4)	0.075	−0.4 (−0.8-−0.03)	0.071
SDAI	−0.005 (−0.02–0.01)	0.55		
Remission, *n* = 19	ref.		ref.	
Low, *n* = 106	**−0.8 (−2–(−0.02))**	**0.044**	−0.8 (−2–0.03)	0.058
Moderate, *n* = 142	**−1 (−2–(−0.3))**	**0.005**	**−1 (−2–(−0.3))**	**0.009**
High, *n* = 40	**−0.9 (−2–(−0.02))**	**0.045**	−0.9 (−2–0.02)	0.054
Moderate and high, *n* = 182	**−1 (−2–(−0.03))**	**0.006**	**−1 (−2–(−0.2))**	**0.011**
CDAI	−0.008 (−0.02–0.02)	0.50		
Remission, *n* = 55	ref.		ref.	
Low, *n* = 137	−0.2 (−0.7–0.3)	0.49	−0.1 (−0.6–0.4)	0.64
Moderate, *n* = 94	**−0.6 (−1–(−0.05))**	**0.031**	−0.5 (−1–(−0.0006))	0.050
High, *n* = 24	−0.1 (−0.9–0.6)	0.76	−0.1 (−0.9–0.6)	0.72
Moderate and high, *n* = 118	−0.5 (−1–0.02)	0.060	−0.5 (−1–0.06))	0.084
Extraarticular manifestations, *n* (%)	−0.3 (−1–0.3)	0.27		
Erosions, *n* (%)	−0.05 (−0.4–0.3)	0.82		
Current drugs, *n* (%)				
Prednisone	−0.007 (−0.4–0.4)	0.97		
Prednisone doses, mg/day	0.05 (−0.06–0.2)	0.36		
NSAIDs	0.02 (−0.3–0.4)	0.92		
DMARDs	−0.4 (−0.8–0.1)	0.13	−0.4 (−0.9–0.09)	0.11
Methotrexate	**−0.5 (−0.8–(−0.09))**	**0.015**	**−0.5 (−0.8–(−0.08))**	**0.018**
Leflunomide	0.4 (−0.09–0.8)	0.12	0.3 (−0.1–0.8)	0.15
Hydroxychloroquine	0.8 (−0.4–2)	0.18	0.9 (−0.3–2)	0.15
Salazopyrin	−1 (−3–1)	0.38		
Anti TNF therapy	0.08 (−0.4–0.6)	0.75		
Tocilizumab	−0.3 (−1–0.5)	0.44		
Rituximab	−0.06 (−1–1)	0.92		
Abatacept	0.4 (−0.9–2)	0.59		
JAK inhibitors	**0.9 (0.1–2)**	**0.022**	**0.9 (0.1–2)**	**0.023**

Data represent mean ± SD or median (IQR) when data were not normally distributed. MPV is considered the dependent variable in this analysis. MPV: Mean platelet volume, fL: femtoliters. NSAID: nonsteroidal anti-inflammatory drugs; DMARD: disease-modifying antirheumatic drug. TNF: tumor necrosis factor. ESR: erythrocyte sedimentation rate, JAK: Janus kinase. BMI: body mass index; DAS28: Disease Activity Score in 28 joints. CRP: C reactive protein. ACPA: anti-citrullinated protein antibodies; CDAI: Clinical Disease Activity Index; SDAI: Simple Disease Activity Index. IL−6: interleukin 6. Dyslipidemia was defined if one of the following was present: total cholesterol > 200 mg/dL, triglycerides > 150 mg/dL, HDL cholesterol < 40 in men or < 50 mg/dL in women, or LDL cholesterol > 130 mg/dL. Multivariable analysis is adjusted for sex and hypertension. Significant *p* values are depicted in bold.

**Table 4:** Relationship of cardiovascular risk parameters to MPV in patients with RA.

		MPV, fL			
		Beta Coefficient (95%CI), *p*			
Carotid ultrasound					
cIMT, mm	0.677 ± 0.131	0.5 (−0.9–2)	0.52		
Carotid plaque, *n* (%)	119 (38)	0.2 (−0.1–0.6)	0.22		
SCORE2					
SCORE2, %	3.2 (1.6–5.6)	−0.01 (−0.06–0.03)	0.64		
Low or moderate risk	216 (70)	ref.		ref.	
High risk	75 (24)	**−0.5 (−0.9–(−0.09)**	**0.018**	**−0.5 (−0.9–(−0.02))**	**0.040**
Very high risk	19 (6)	0.3 (−0,2–0.8)	0.25	0.3 (−0.2–0.9)	0.21
Lipid profile					
Total cholesterol, mg/dL	206 ± 37	−0.001 (−0.006–0.004)	0.66		
Triglycerides, mg/dL	153 ± 90	−0.0004 (−0.002–0.002)	0.68		
HDL-cholesterol, mg/dL	57 ± 15	0.005 (−0.007–0.02)	0.40		
LDL-cholesterol, mg/dL	119 ± 34	−0.002 (−0.007–0.004)	0.52		
LDL:HDL cholesterol ratio	2.27 ± 0.96	−0.2 (−0.3–0.03)	0.10	−0.1 (−0.3–0.06)	0.19
Non-HDL cholesterol, mg/dL	150 ± 38	−0.002 (−0.007–0.003)	0.44		
Lipoprotein (a), mg/dL	33 (11–103)	−0.001 (−0.003–0.001)	0.41		
Apolipoprotein A1, mg/dL	174 ± 29	0.005 (−0.002–0.01)	0.14		
Apolipoprotein B, mg/dL	108 ± 48	−0.002 (−0.006–0.002)	0.26		
Apo B:Apo A1 ratio	0.63 ± 0.25	−0.07 (−1–0.04)	0.063	−0.6 (−1–0.1)	0.10
Atherogenic index	3.91 ± 1.35	−0.1 (−0.2–0.03)	0.12	−0.08 (−0.2–0.06)	0.25
Insulin resistance indices					
Insulin, µU/ml	7.7 (5.1–13.3)	0.01 (−0.007–0.02)	0.21		
C-peptide, ng/ml	3.1 ± 2.3	0.03 (−0.06–0.1)	0.50		
HOMA2-IR	0.99 (0.64–1.72)	0.1 (−0.05–0.3)	0.20		
HOMA2-S%	100 (58–156)	0.001 (−0.001–0.003)	0.39		
HOMA2-B%-C-peptide	174 ± 77	−0.0008 (−0.003–0.002)	0.55		

Data represent mean ± SD or median (IQR) when data were not normally distributed. PPV is the dependent variable. Insulin resistance analysis is only performed for non-diabetic patients and if glucose is lower than 110 mg/dL (*n* = 265). SCORE: Systematic Coronary Risk Evaluation, LDL: low-density lipoprotein; HDL: high-density lipoprotein. cIMT: carotid intima media thickness. HOMA: homeostatic model assessment, CI: confidence interval. MPV: mean platelet volume, fL: femtoliters. Multivariable analysis is adjusted for sex and hypertension (hypertension is not included in the SCORE2 adjustment). Significant p value are depicted in bold.

## Data Availability

The data sets used and/or analyzed in the present study are available from the corresponding author upon request.

## Abbreviations

ACPA: Anti-citrullinated protein antibodies; BMI: body mass index; carotid ultrasound was not available for controls. CDAI: Clinical Disease Activity Index; cIMT: carotid intima media thickness; CRP: C reactive protein; CV: cardiovascular; DAS28: Disease Activity Score in 28 joints; DMARD: disease-modifying antirheumatic drug; ESR: erythrocyte sedimentation rate; fL: femtoliters; HDL: high-density lipoprotein; HOMA: homeostatic model assessment; IL: interleukin; IQR: interquartile range; JAK: Janus kinase; LDL: low-density lipoprotein; MPV: mean platelet volume; NSAID: nonsteroidal anti-inflammatory drugs; TNF: tumor necrosis factor; obesity; RA: rheumatoid arthritis; SCORE: Systematic Coronary Risk Evaluation; SDAI: Simple Disease Activity Index.
